# Supplementary material for: A mathematical model of fibrinogen-mediated erythrocyte–erythrocyte adhesion
Source: Commun Biol. 2023 Feb 17;6:192. doi: 10.1038/s42003-023-04560-4 (PMC9938206; doi:10.1038/s42003-023-04560-4)
Supplement: Supplementary file 2 — Description of Additional Supplementary Files [file 42003_2023_4560_MOESM2_ESM.pdf]

Description of Additional Supplementary Files

File name: Supplementary Data
Description: Data for Figs 2-3 and code for Figs 4-6.
