# Supplementary material for: A mathematical model of fibrinogen-mediated erythrocyte–erythrocyte adhesion
Source: Commun Biol. 2023 Feb 17;6:192. doi: 10.1038/s42003-023-04560-4 (PMC9938206; doi:10.1038/s42003-023-04560-4)
Supplement: Supplementary file 4 — Reporting Summary [file 42003_2023_4560_MOESM4_ESM.pdf]

nature portfolio

Corresponding author(s): Rui D.M. Travasso / Nuno C. Santos

Last updated by author(s): Jan 18, 2023

Reporting Summary

Nature Portfolio wishes to improve the reproducibility of the work that we publish. This form provides structure for consistency and transparency in reporting. For further information on Nature Portfolio policies, see our Editorial Policies and the Editorial Policy Checklist.

Statistics

For all statistical analyses, confirm that the following items are present in the figure legend, table legend, main text, or Methods section.

n/a	Confirmed	
☐	☒	The exact sample size (*n*) for each experimental group/condition, given as a discrete number and unit of measurement
☐	☒	A statement on whether measurements were taken from distinct samples or whether the same sample was measured repeatedly
☐	☒	The statistical test(s) used AND whether they are one- or two-sided<br>*Only common tests should be described solely by name; describe more complex techniques in the Methods section.*
☒	☐	A description of all covariates tested
☒	☐	A description of any assumptions or corrections, such as tests of normality and adjustment for multiple comparisons
☐	☒	A full description of the statistical parameters including central tendency (e.g. means) or other basic estimates (e.g. regression coefficient) AND variation (e.g. standard deviation) or associated estimates of uncertainty (e.g. confidence intervals)
☒	☐	For null hypothesis testing, the test statistic (e.g. *F*, *t*, *r*) with confidence intervals, effect sizes, degrees of freedom and *P* value noted<br>*Give P values as exact values whenever suitable.*
☒	☐	For Bayesian analysis, information on the choice of priors and Markov chain Monte Carlo settings
☒	☐	For hierarchical and complex designs, identification of the appropriate level for tests and full reporting of outcomes
☒	☐	Estimates of effect sizes (e.g. Cohen's *d*, Pearson's *r*), indicating how they were calculated

Our web collection on statistics for biologists contains articles on many of the points above.

Software and code

Policy information about availability of computer code

Data collection	JPK data analysis software (JPK Instruments) and home-built LabVIEW program
Data analysis	JPK data processing software version 6.0.55 (JPK Instruments); Graphpad Prism version 8.0; ImageJ software version 1.53e (NIH,USA); home-built code to integrate differential equations.

For manuscripts utilizing custom algorithms or software that are central to the research but not yet described in published literature, software must be made available to editors and reviewers. We strongly encourage code deposition in a community repository (e.g. GitHub). See the Nature Portfolio guidelines for submitting code & software for further information.

Data

Policy information about availability of data

All manuscripts must include a data availability statement. This statement should provide the following information, where applicable:

- Accession codes, unique identifiers, or web links for publicly available datasets
- A description of any restrictions on data availability
- For clinical datasets or third party data, please ensure that the statement adheres to our policy

The data that is plotted in Figs 2 and 3 of this study are available from the corresponding authors upon reasonable request.

Human research participants

Policy information about studies involving human research participants and Sex and Gender in Research.

Reporting on sex and gender	This information was not collected
Population characteristics	All the participants were healthy volunteer blood donors
Recruitment	Five healthy volunteer blood donors were included in this study to perform the micropipette aspiration technique, following a protocol with Odense University Hospital (Denmark). For the AFM studies, we used a control group of 28 healthy blood donors, following a protocol with Instituto Português do Sangue e da Transplantação (IPST, Lisbon, Portugal).
Ethics oversight	Ethical committee of Odense University Hospital, University of Southern Denmark (SDU), and Joint Ethics Committee of Faculdade de Medicina da Universidade de Lisboa and Centro Hospitalar Universitário Lisboa Norte.

Note that full information on the approval of the study protocol must also be provided in the manuscript.

Field-specific reporting

Please select the one below that is the best fit for your research. If you are not sure, read the appropriate sections before making your selection.

☒ Life sciences ☐ Behavioural & social sciences ☐ Ecological, evolutionary & environmental sciences

For a reference copy of the document with all sections, see nature.com/documents/nr-reporting-summary-flat.pdf

Life sciences study design

All studies must disclose on these points even when the disclosure is negative.

Sample size	Blood from five donors for the micropipette aspiration technique and 28 for the AFM studies
Data exclusions	No data were excluded
Replication	The total number of erythrocyte pairs analyzed were 32 and 34, with and without fibrinogen addition, respectively. For each erythrocyte pair, we obtained 16 ± 5 adhesion image sequences, with a 15s cycle each, and determined if cell-cell adhesion was observed. For AFM, on each blood sample, approximately 40 force-distance curves were performed for each concentration.
Randomization	The blood was collected on the day each volunteer went randomly to give blood at Odense Hospital or IPST (Lisbon). Healthy donors were not specifically selected for this study.
Blinding	The research work is in compliance with the EU regulations for processing of personal data and on the free movement of such data (EU Regulation 2016/679). All the information related to individuals was managed after explicit consent and in compliance with the European legislations. Procedures of data collection comply with the applicable international, EU and National laws (in particular, EU Directive 95/46/EC) in order to limit the impact on the enrolled participants and ensure data quality and confidentiality. We used the internal data storage service of iMM, which provides a secure place to keep our data. After coding the individual identification, data was transposed into an electronic format, and the coding key will only be known to the researcher accompanying the study. This project ensures anonymity and confidentiality of data and samples during all operations. It also guarantees that samples comply with scientific and ethical requirements, after previous external approval by the Joint Ethics Committee of Faculdade de Medicina da Universidade de Lisboa and Centro Hospitalar Universitário Lisboa Norte.

Reporting for specific materials, systems and methods

We require information from authors about some types of materials, experimental systems and methods used in many studies. Here, indicate whether each material, system or method listed is relevant to your study. If you are not sure if a list item applies to your research, read the appropriate section before selecting a response.

Materials & experimental systems

n/a	Involved in the study
☒	☐ Antibodies
☒	☐ Eukaryotic cell lines
☒	☐ Palaeontology and archaeology
☒	☐ Animals and other organisms
☒	☐ Clinical data
☒	☐ Dual use research of concern

Methods

n/a	Involved in the study
☒	☐ ChIP-seq
☒	☐ Flow cytometry
☒	☐ MRI-based neuroimaging
